# Inter-rater reliability of paediatric emergency assessment: physiological and clinical features

**DOI:** 10.1136/archdischild-2019-318664

**Published:** 2020-09-28

**Authors:** Calvin Heal, Sarah Cotterill, Andrew Graeme Rowland, Natalie Garratt, Tony Long, Stephen Brown, Grainne O'Connor, Chloe Rishton, Steve Woby, Damian Roland

**Affiliations:** 1 Centre for Biostatistics, The University of Manchester Faculty of Biology Medicine and Health, Manchester, UK; 2 School of Health and Society, The University of Salford, Salford, UK; 3 Emergency Department, North Manchester General Hospital, Manchester, UK; 4 Northern Care Alliance NHS Group, Salford, UK; 5 Paediatric Emergency Medicine Leicester Academic (PEMLA) Group, Leicester Royal Infirmary, Leicester, UK; 6 SAPHIRE Group, University of Leicester, Leicester, UK

**Keywords:** paediatric practice, general paediatrics, accident & emergency

## Abstract

**Objective:**

The Paediatric Admission Guidance in the Emergency Department (PAGE) score is an assessment tool currently in development that helps predict hospital admission using components including patient characteristics, vital signs (heart rate, temperature, respiratory rate and oxygen saturation) and clinical features (eg, breathing, behaviour and nurse judgement). It aims to assist in safe admission and discharge decision making in environments such as emergency departments and urgent care centres. Determining the inter-rater reliability of scoring tools such as PAGE can be difficult. The aim of this study was to determine the inter-rater reliability of seven clinical components of the PAGE Score.

**Design:**

Inter-rater reliability was measured by each patient having their clinical components recorded by two separate raters in succession. The first rater was the assessing nurse, and the second rater was a research nurse.

**Setting:**

Two emergency departments and one urgent care centre in the North West of England. Measurements were recorded over 1 week; data were collected for half a day at each of the three sites.

**Patients:**

A convenience sample of 90 paediatric attendees (aged 0–16 years), 30 from each of the three sites.

**Main outcome measures:**

Two independent measures for each child were compared using kappa or prevalence-adjusted bias-adjusted kappa (PABAK). Bland-Altman plots were also constructed for continuous measurements.

**Results:**

Inter-rater reliability ranged from moderate (0.62 (95% CI 0.48 to 0.74) weighted kappa) to very good (0.98 (95% CI 95 to 0.99) weighted kappa) for all measurements except ‘nurse judgement’ for which agreement was fair (0.30, 95% CI 0.09 to 0.50 PABAK). Complete information from both raters on all the clinical components of the PAGE score were available for 73 children (81%). These total scores showed good’ inter-rater reliability (0.64 (95% CI 0.53 to 0.74) weighted kappa).

**Conclusions:**

Our findings suggest different nurses would demonstrate good inter-rater reliability when collecting acute assessments needed for the PAGE score, reinforcing the applicability of the tool. The importance of determining reliability in scoring systems is highlighted and a suitable methodology was presented.

## Abstract (precis)

This study assesses the inter-rater reliability of the Paediatric Admission Guidance in the Emergency Department (PAGE) score. The PAGE score is an assessment tool currently in development that helps predict hospital admission using components including patient characteristics, vital signs and clinical features, to assist in safe admission and discharge. The authors found good inter-rater reliability among a group of nurses using the tool.

**What is already known on this topic?**

- Early warning scores in emergency departments are becoming increasingly popular, but their inter-rater reliability is generally poorly understood.
- The current most popular bespoke early warning score, POPS, has good inter-rater reliability.

**What this study adds?**

- Inter-rater reliability of the vital signs and clinical features that may make up the Paediatric Admission Guidance in the Emergency Department (PAGE) score are generally good.
- Resultantly, the inter-rater reliability of the PAGE score appears to be good based on the researchers’ retrospective calculation of the total score.
- A ‘second rater’ is a feasible and easily applied method of measuring inter-rater reliability.

## Introduction

While triage remains an important component of emergency care practice, the importance of an early full assessment of physiology and behaviour is increasingly recognised. In the UK, national guidance suggests all children attending emergency care settings should be visually assessed by a registered practitioner immediately on arrival with clinical assessment undertaken within 15 min to determine priority category, supplemented by a pain score and a full record of vital signs.^1^

There has been a trend towards creating composite scores from these assessments to allow a global measure of acuity to be determined. These are commonly referred to as Early Warning Scores or Track and Trigger systems^2^ and generally assign a numeric value to a range of vital signs or observational characteristics (eg, heart rate within the normal range for a child of a certain age receives a score of 0, heart rate above (or below) the normal range receives 1 and heart rate significantly above (or below) the normal range receives 2). Traditionally, these systems have been developed and validated in inpatient ward settings. Their introduction into emergency departments (EDs) is a relatively new occurrence, and there have been concerns with their sensitivity and specificity.^3^ For this reason, bespoke emergency care assessment systems have been created, but while the effectiveness of these systems at determining disposition (the location to which the child goes when discharged from the ED) and clinical outcome is subject to considerable academic scrutiny, the inter-rater reliability of the use of such scoring systems has been poorly examined.

In the UK, the Paediatric Observation Priority Score (POPS) is the most commonly used bespoke early warning score in EDs.^4^ When examined using previously recorded video assessments of children, POPS has been shown to have ‘perfect’ inter-rater reliability for well children and ‘good’ inter-rater reliability with an intraclass correlation coefficient (ICC) between 0.66 and 0.74 for unwell children.^5^

A subsequent study with a larger sample of children (n=11 videos) and raters (n=46) found a high overall ICC between the raters (95% CI 0.71 to 0.95).^6^ On individual components of POPS, the study found high rates of agreement (Fleiss’ kappa) for oxygen saturation (over 0.87) and pulse (over 0.76). Agreement was lower and more variable on work of breathing (0.48–0.91), gut feeling (0.45–0.87), conscious level (0.55–1), medical history (0.53–1), respiratory rate (0.44–0.96) and temperature (0.51–1). A limitation of these works however was the use of video recordings that do not give a complete contextual picture of the child.

The Pennine Acute Hospitals NHS Trust Paediatric Observation Priority Score (PAT-POPS) is a modified version of the original POPS and is a specific ED physiological and observational aggregate scoring system, with scores of 0–18. A higher score indicates greater likelihood of admission. PAT-POPS has been shown to be a more accurate predictor of admission risk than the Manchester Children's Early Warning System, which assesses six physiological observations to create a trigger score, classified as green, amber or red.^7^ The inter-rater reliability of PAT-POPS has never previously been studied.

As part of a National Institute for Health Research Research for Patient Benefit study (the host study), the current PAT-POPS tool has been expanded and refined to improve diagnostic accuracy. The protocol for this work has been published.^8^ This work has led to the development of a Paediatric Admission Guidance in the Emergency department (PAGE) score based on data from 44 501 paediatric (children under 16 years) visits to three EDs and an urgent care centre within North West England. The aim of this sub-study was to examine real-time reliability of the vital sign and clinical observation components of the score by assessing the degree to which they can be reliably recorded by two nurses, and the extent to which there is agreement on the overall PAGE score as calculated retrospectively by this report’s authors. This provides another measure of reliability and helps highlight potential components of the system that may not be reproducible across observers.

## Method

### Setting

Data for the host study were collected from all children aged under 16 years attending two EDs and one urgent care centre during a 12-month period (21 February 2018–21 February 2019) in the North West of England. Patients were recruited using an opt-out process that informed them of the parent study. The research nurses verbally explained the nature and purpose of the inter-rater reliability data collection. Based on previous research, a number of vital signs and clinical observations were obtained from all children including heart rate, temperature, respiratory rate, oxygen saturation, behaviour, work of breathing and nurse judgement (overall concern). Other components that were part of the derivation components of the PAGE score were age, presence of pre-existing morbidity, arrival by ambulance and advice by a medical professional to attend, but these were not formally collected by the assessing nurse.

### Raters and data collection

Independent double data collection was arranged during 1 week. An additional rater visited each of the three sites for half a day and completed a full set of measurements needed for the PAGE score. The first rater was the person who would normally do the observations at that hospital site (the assessing nurse), and they recorded the data via the study protocol: entering the raw observations on the hospitals information technology system. This is the system the staff used in their normal clinical practice so no training for this was required. The data for the inter-rater reliability component were collected from three hospital sites and by all nurses at those sites, so there were multiple ‘first raters’. The second rater was a single research nurse with a paediatric background, used throughout the study. After being briefed on the procedures, the second rater recorded their measurements on a different information database. The two assessments were undertaken as simultaneously as possible with the time frame between them being no more than 10 min. The study’s database manager matched the two entries by locating patients using their unique hospital number. Although the observations were not shared between the two raters, nor were they explicitly blinded to each other’s observations.

Dual observations were completed on 30 children per site (n=90 in total across the three sites). The children were chosen as a convenience sample.

### Analysis

The inter-rater reliability of each variable is reported separately. For each variable, the raw values between the two raters were compared. Ordinal variables and continuous variables without a normal distribution were assessed using a weighted kappa. Where the distribution of frequencies was so uneven as to cause excessive bias in the kappa scores, the prevalence-adjusted and bias-adjusted kappa (PABAK) was reported instead. The reliability of continuous variables was also examined with Bland-Altman plots and accompanying limits of agreement. CIs were derived using bootstrapping (2000 repetitions) for weighted kappa and jackknife SEs for PABAK. In interpreting these results, the guidelines from Altman^9^ were used, as shown in table 1.

**Table 1:** Guidelines for interpreting kappa values

Kappa value	Strength of agreement
<0.00	–
0.00–0.20	Poor
0.21–0.40	Fair
0.41–0.60	Moderate
0.61–0.80	Good
0.81–1.00	Very good

Each child’s PAGE score was calculated for both raters (ie, the score created if a numerical value was assigned to patient characteristics, vital signs and clinical features). This was done using a formula in Stata, eliminating the possibility of error in calculating the score. Reliability of the composite PAGE score was assessed with a weighted kappa. This was undertaken for those patients who had all the information required to calculate a PAGE score, and as a sensitivity analysis, a PAGE score was calculated in the presence of missingness as would likely happen in practice. When data for a variable were missing, it was scored as 0. As 0 is a valid value for each of the variables, however, doing so may artificially increase disagreement.

## Results

Data were collected from 90 patients. Two patients had missing or mismatched participant IDs and so could not be included in the analysis. The reliability and 95% CI for each PAGE variable, for the remaining 88 patients, are summarised in table 2. For each variable, there were some (between zero and eight) participants who had a missing value so the number included in the assessment is stated in the ‘n’ column.

**Table 2:** Results of inter-rater reliability for each variable

Variable	n	Reliability	95% CI	Method
Heart rate (bpm)	87	0.84	0.80 to 0.89	Weighted kappa
Temperature (°C)	80	0.98	0.95 to 0.99	Weighted kappa
Respiratory rate (breaths per min)	86	0.71	0.62 to 0.79	Weighted kappa
Oxygen saturation (%)	87	0.62	0.48 to 0.74	Weighted kappa
Behaviour (normal for age, agitated, floppy, listless and inappropriate)	88	0.92	0.86 to 0.99	PABAK
Breathing (audible grunt, stridor, tracheal tug, wheeze and none of the above)	88	0.97	0.92 to 1.00	PABAK
Nurse judgement (no judgement, low-level concern and child looks unwell/high-level concern)	88	0.30	0.09 to 0.50	PABAK
Total PAGE score (retrospectively calculated)	73	0.64	0.53 to 0.73	Weighted kappa

PABAK, prevalence-adjusted and bias-adjusted kappa; PAGE, Paediatric Admission Guidance in the Emergency department.

The variables temperature (0.98 (95% CI 0.95 to 0.99) weighted kappa), breathing (0.97 (95% CI 0.92 to 1.00) PABAK) and behaviour (0.92 (95% CI 0.86 to 0.99) PABAK) exhibited excellent reliability. With breathing and behaviour, it should be noted that the relative rarity of responses outside the normal clinical range undoubtedly helped improve reliability. The reliability of heart rate was ‘very good’ (0.84 (95% CI 0.80 to 0.89) weighted kappa). The reliability of respiratory rate (0.71 (95% CI 0.62 to 0.79) weighted kappa) and oxygen saturation (0.62 (95% CI 0.48 to 0.74) weighted kappa) can be considered ‘good’ and ‘moderate’, respectively. The only measure with ‘fair’ agreement is nurse judgement (0.30 (95% CI 0.09 to 0.50) PABAK), and this is broken down in table 3.

**Table 3:** Frequency of nurse judgement values selected by each rater

		Second rater			
		High-level concern	Low-level concern	No concern	Total
First rater	High-level concern	0	0	0	0
	Low level concern	0	2	29	31
	No concern	0	2	55	57
	Total	0	4	84	88

When the two raters were asked to make an overall nurse judgement about the child’s health, the first rater classified 31 out of 88 children as low concern, whereas the second rater considered 29 of these 31 children of no concern. This suggests that the raters were unable to reliably agree on a distinction between no concern and low level concern. There were no children in this sample that were rated as ‘High concern’ by either of the raters, so it is not possible to understand whether the raters can reliably differentiate ‘no/low concern’ children from ‘high concern’ children.

**Figure 1:**
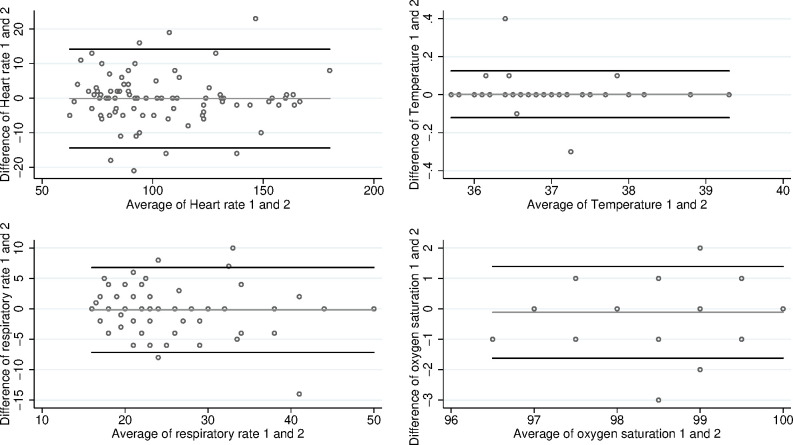
Bland-Altman plots for continuous variables.

Bland-Altman plots are shown in figure 1. The 95% limits of agreement (and accompanying 95% CIs) for heart rate are −14.1 (−16.7 to −11.5) to 13.9 (11.3 to 16.5). For temperature, they are −0.1 (−0.1 to −0.1) to 0.1 (0.1 to 0.1), for respiratory rate they are −7.0 (−8.3 to −5.8) to 6.7 (5.4 to 7.9) and for oxygen saturation they are −1.6 (−1.9 to −1.4) to 1.4 (1.1 to 1.7).

A total PAGE score was calculated for each patient, separately for each of the two raters. Of the 88 patients, 73 (83%) had complete information from both raters for each of the 13 variables required to compute PAGE (the seven vital variables observed by the raters plus other variables collected at reception including personal characteristics, arrival by ambulance and referral source). These total scores showed ‘good’ inter-rater reliability (0.64 (95% CI 0.53 to 0.73) weighted kappa). As the tool had not been finalised, the total scores were not calculated by the research nurses but were calculated later by this report’s authors. As a sensitivity analysis, the PAGE score was also computed where there was some missingness (when not all of the 13 items of the PAGE score had a score recorded), the inter-rater reliability of the total score remained essentially unchanged: a 0.64 (95% CI 0.59 to 0.74) weighted kappa score.

## Discussion

The inter-rater reliability of vital sign and clinical observation scores by two separate observers was generally found to be good. Temperature, breathing and behaviour were recorded with a very high level of agreement between the two nurses. Heart rate, respiratory rate and oxygen saturation saw good or very good levels of agreement. However, only a fair degree of agreement was achieved between the two nurses on the nurse judgement variable, at least between the ‘no’ and ‘low’ levels of concern. That this study failed to show agreement between ‘no’ and ‘low’ level concern should not preclude the variable’s inclusion in the PAGE score. There is reason to believe that the distinction between ‘high’ and ‘no’/’low’ concern will be more clinically important than the distinction between ‘low’ and ‘none’ though as we had no ‘high’ concern patients, there is no evidence available regarding this.

Regarding the total PAGE score that would arise from the assessments made by the two raters, calculated retrospectively by the authors, there is some variability here too as would be expected. The ‘good’ inter-rater reliability of the total score however reflects reasonably well the overall state of agreement between the nurses. A limitation of the study is that the score was calculated using software, whereas in clinical practices, there may be additional sources of variability, such as summing errors or other unanticipated influences on the scoring of each variable.

This is the first time a study of the reliability of an ED scoring system has been undertaken with this methodology. The fact that inter-rater reliability work is rarely performed with scoring systems is highlighted, and this is a useful methodology in conjunction with the video approach taken in the previous POPS studies.^5 6^ The variability in the inter-rater reliability of the various measures recorded is likely due to a combination of either slight differences in timings of assessments between the assessor, differences in technique of the nurses and differences in subjective assessment based on experience or expectations. Although observations were taken within the same triage assessment time, the state of the child could vary significantly within this time frame. One example of this occurring was one nurse taking the readings when the child was settled and the other nurse taking them following a blood pressure measurement, when the child was upset. The method of assessing video recordings of paediatric assessments to measure inter-rater reliability, used previously for POPS, fails to capture much of this variability. Using a second rater more accurately captures the real-life variability of paediatric assessments, although not without its own limitations. Aside from being very time consuming, the nurses involved in the data collection also raised the point that they felt that being observed (by the other nurse) may have influenced their behaviour. This may have then influenced the measurements they recorded: a potential limitation of any study where participant’s behaviour is directly observed but one that should still be recognised.

Another limitation of the study is that of the 90 children involved, none were of ‘high concern’ to the nurses. As a result, although there is no reason to suspect it would differ, this cannot speak for the inter-rater reliability of nurse measurements for very unwell or severely injured children. This is an important limitation as the reliability of the components of any score that is aiming to differentiate sick from non-sick children must be effective for both sick and non-sick children. Specific studies may be needed focusing only on those children who are in small percentage of cases who require urgent intervention to ensure the score robustly identifies them. Previous large data sets have demonstrated that clinical signs tend to converge together and that is unlikely there will be large differences in interpretation for this most sick cohort even if some data are missing.^10^

## Conclusion

Although difficult to compare directly, due to the different study designs and agreement measurements used, it seems the overall inter-rater reliability of the measures that make up vital signs and clinical observation criteria is at least as good as POPS and sufficient for wider use. There are several elements of subjectivity inherent in any ED point scoring system completed by humans, but it seems these result in an acceptable amount of variation. The use of the direct observation methodology described in this paper is highlighted as possibly methodology for other studies of scoring systems.
